# Regional variations in gynecological hospitalization patterns in Northeastern China: a cross-sectional analysis of 4,935 inpatients with implications for nursing practice

**DOI:** 10.3389/frph.2026.1804419

**Published:** 2026-04-20

**Authors:** Jingjing Tang, Haitao Wang

**Affiliations:** 1 Department of Gynecology, Jixi Jikuang Hospital, Jixi, Heilongjiang, China; 2 School of Resource Engineering, Heilongjiang University of Technology, Jixi, China

**Keywords:** cross-sectional study, gynecological diseases, hospitalization patterns, mining region, nursing implications, regional epidemiology

## Abstract

**Purpose:**

This study analyzed the epidemiological characteristics and distribution patterns of gynecological diseases among hospitalized patients in a tertiary hospital serving China's northeastern mining region, compared disease profiles with national averages, and discussed implications for targeted nursing interventions.

**Methods:**

A retrospective cross-sectional study was conducted using electronic medical records from 4,935 gynecological inpatients at Jixi Jikuang Hospital, Heilongjiang Province (January 2021–December 2025). Data collected included age, primary diagnosis (ICD-10 classification), residential district, insurance type, and length of stay. Regional hospitalization rates were calculated using Seventh National Census population data. Chi-square tests compared disease proportions with published national averages.

**Results:**

Analysis of 4,935 patients revealed distinct epidemiological patterns. The majority (60.7%) were aged 40–59 years, with peak prevalence observed in the 40–49 age group (33.8%; mean age 47.2 ± 12.8 years). Uterine pathologies dominated disease composition (57.6%), primarily endometrial polyps (18.9%) and uterine fibroids (18.6%), followed by ovarian diseases (15.9%) and cervical pathologies (13.5%). Geospatial analysis identified that hospitalization rates in mining-intensive districts (518.9–914.3 per 100,000) were higher compared with non-mining areas (178.2–207.8 per 100,000). Most hospitalizations lasted 3–8 days (66.2% of cases). Compared with national averages, significantly higher proportions were observed for endometrial polyps (18.9% vs. 14.5%, *P* < 0.001), uterine fibroids (18.6% vs. 15.2%, *P* < 0.001), and cervical neoplasms (10.2% vs. 7.8%, *P* < 0.001).

**Conclusion:**

Gynecological hospitalizations in this northeastern mining region exhibit clustering in perimenopausal women, elevated uterine pathology prevalence, and geographic disparities favoring mining-intensive districts. These findings suggest potential environmental or healthcare access factors, warranting further investigation, and support the development of region-specific, age-stratified nursing care models.

## 1 Introduction

Gynecological diseases constitute a critical public health concern, affecting women's health worldwide. According to World Health Organization estimates, approximately 30% of reproductive-aged women suffer from gynecological disorders of varying severity, significantly impairing quality of life and reproductive health (1–3). In China, the burden of gynecological diseases is particularly pronounced, with the 2020 National Women's Health Survey revealing an overall prevalence of 41.2%, with uterine fibroids, cervical pathologies, and ovarian cysts ranking as the most common conditions (4).

**Figure 1:**
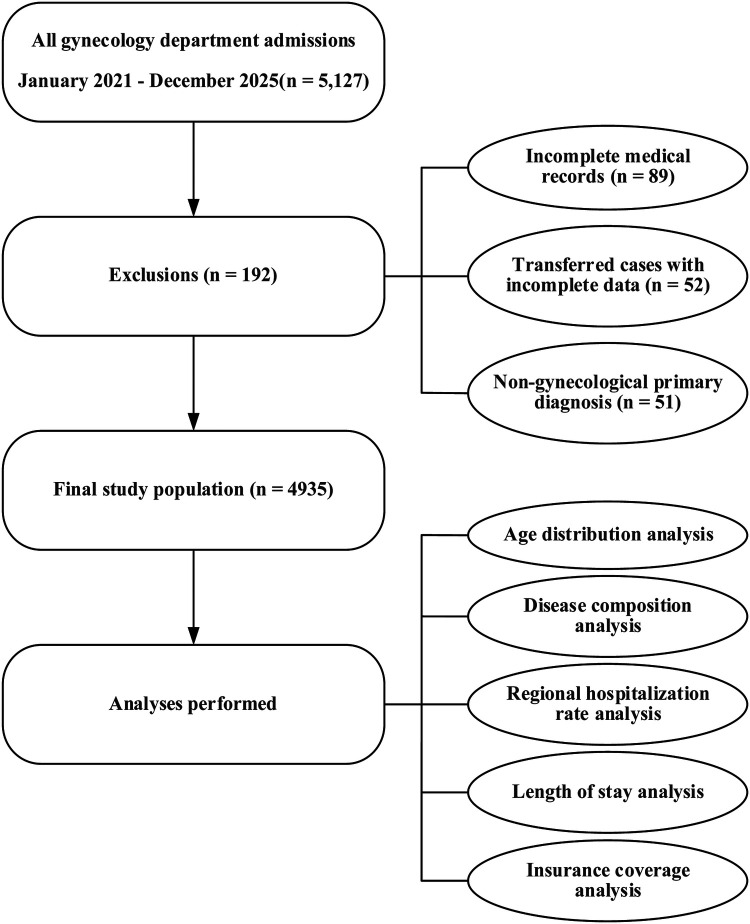
Patient selection flow chart showing inclusion and exclusion of study participants.

Regional variations in disease patterns have been documented globally, influenced by environmental exposures, healthcare access, socioeconomic conditions, and lifestyle factors (5). Resource-dependent regions, particularly mining areas, present unique public health challenges. Previous epidemiological studies have suggested that populations residing in mining regions may face elevated risks of certain health conditions due to environmental contamination, occupational hazards, and associated socioeconomic factors (6). In the context of women's reproductive health, studies from various mining regions have reported associations between environmental exposures and adverse reproductive outcomes, though evidence specifically linking mining-area residence to gynecological disease patterns remains limited (7).

Jixi City in Heilongjiang Province represents a typical coal-resource-dependent urban center in northeastern China. The region's demographic characteristics—including an aging population structure, industrial economic base, and distinct climatic conditions—may contribute to unique patterns of gynecological disease burden (8). However, systematic epidemiological data characterizing gynecological hospitalization patterns in this region are lacking, limiting the ability to develop evidence-based, region-specific healthcare strategies.

Understanding regional disease patterns is essential for optimizing healthcare resource allocation and nursing care delivery. Nursing plays a pivotal role in gynecological disease management, encompassing perioperative care, patient education, symptom management, and continuity of care (9). The development of nursing strategies tailored to regional disease profiles and patient demographics represents an important component of precision healthcare delivery (10). However, such tailoring requires comprehensive epidemiological data that are currently unavailable for many regions in China.

In light of these knowledge gaps, this retrospective cross-sectional study was designed to (1) characterize the demographic and clinical features of gynecological inpatients at a tertiary hospital in Jixi City; (2) analyze disease composition and compare proportions with national averages; (3) examine geographic variations in hospitalization rates across the region's administrative districts; and (4) discuss implications for nursing practice and healthcare planning. The findings aim to provide foundational epidemiological data for this understudied region and inform the development of targeted healthcare strategies.

## 2 Materials and methods

### 2.1 Study design and setting

This retrospective, cross-sectional study was conducted at Jixi Jikuang Hospital, a 500-bed tertiary hospital and the main referral center for gynecologic care in the Jixi mining region of Heilongjiang Province, northeastern China. The hospital serves six urban districts (Jiguan, Hengshan, Didao, Chengzihe, Lishu, and Mashan), one county (Jidong), and two county-level cities (Mishan and Hulin), covering approximately 1.5 million residents according to the Seventh National Census (2020) (11).

### 2.2 Study population

This retrospective cross-sectional study analyzed electronic medical records of all female patients admitted to the gynecology department between 1 January 2021 and 31 December 2025, using consecutive sampling of all eligible patients to minimize selection bias.

Inclusion criteria were defined as follows. Eligible cases were those involving patients hospitalized under gynecological care with complete documentation of key demographic variables, including age and place of residence, as well as a physician-confirmed primary gynecologic diagnosis. In addition, all included admissions met the World Health Organization's adolescent health guidelines and conformed to the institutional protocols governing the initiation of gynecological care.

Exclusion criteria were applied in a standardized manner. Records were excluded if essential diagnostic or geographic information was missing, or if the patient was transferred between hospitals, resulting in incomplete documentation of the index hospitalization at the study site. Admissions in which gynecologic conditions were recorded only as incidental findings, and in which the primary reason for hospitalization was non-gynecologic in nature, were also excluded, as confirmed through review of the corresponding ICD-10 codes. A flowchart detailing the patient selection process is provided in Figure 1.

### 2.3 Data collection

Data were extracted from the electronic medical record system by trained research staff using a standardized form. Extracted variables included age at admission, residence (classified by registered address), insurance type (urban employee, urban–rural resident, self-pay, or other), primary discharge diagnosis (ICD-10), and length of hospital stay (days).

Primary discharge diagnoses were categorized into major gynecologic disease groups (uterine, ovarian, cervical, fallopian tube, ectopic pregnancy, pelvic, vaginal, abortion-related, and other disorders) and further subcategorized.

#### 2.3.1 Limitations of data source

As this study relied exclusively on electronic medical records, certain variables relevant to understanding disease patterns were not available. These included detailed occupational history, duration of residence in current location, specific environmental or occupational exposures, lifestyle factors (diet, physical activity, smoking), and reproductive history details. These limitations are acknowledged in the Study Limitations section.

### 2.4 Geographic classification

Patients were classified by residential district based on their registered address. For calculation of regional hospitalization rates, districts were classified as “mining-intensive” (Mashan, Hengshan, Chengzihe, and Didao) or “non-mining/mixed economy” (Jiguan, Lishu, Jidong, Mishan, and Hulin), based on the predominant local economic activities and historical mining operations documented in regional economic reports.

### 2.5 Comparison with national data

To contextualize regional findings, disease proportions were compared with national averages derived from the National Women's Health Statistical Yearbook (2020–2023) published by the National Health Commission of China (12). This source provides aggregated data on gynecological disease composition from tertiary hospitals across China, enabling standardized comparison.

### 2.6 Statistical analysis

Data management and analysis were performed using SPSS and Origin software. Continuous variables were expressed as mean ± standard deviation (SD) and compared using independent-sample *t*-tests (two groups) or one-way analysis of variance (multiple groups). Categorical variables were presented as frequencies and percentages.

Regional hospitalization rates were calculated as follows:

$$\begin{array}{rl} & Hospitalization\;rate\\ & =\;(Number\;of\;patients\;from\;region\;/\;Regional\;population)\times 100,000 \end{array}$$

Chi-square goodness-of-fit tests were used to compare observed disease proportions in our sample with expected proportions based on national averages. For these comparisons, the null hypothesis stated that our sample's disease distribution matched the national distribution. The relationship between age and length of hospital stay was evaluated using segmented linear regression analysis, with coefficient of determination (*R*^2^) assessing model fit. All statistical tests were two-tailed, with significance threshold set at *P* < 0.05.

## 3 Results

### 3.1 Patient characteristics

A total of 4,935 gynecological inpatients met the inclusion criteria and were included in the final analysis. Patient demographic and clinical characteristics are summarized in Table 1.

**Table 1:** Demographic and clinical characteristics of gynecological patients (*n* = 4,935).

Category	Group/indicator	Number (*n*)	Proportion
Age distribution			
	8–19 years	66	1.3%
	20–29 years	243	4.9%
	30–39 years	857	17.4%
	40–49 years	1,669	33.8%
	50–59 years	1,327	26.9%
	60–69 years	507	10.3%
	70–79 years	217	4.4%
	≥80 years	49	1.0%
Length of stay			
	1–2 days	250	5.1%
	3–5 days	1,732	35.1%
	6–8 days	1,534	31.1%
	9–14 days	1,032	20.9%
	15–30 days	370	7.5%
	≥31 days	17	0.3%
Medical insurance			
	Jixi Urban Employee	1,881	38.0%
	Jixi Urban–Rural Resident	1,594	32.3%
	Jidong County Employee	76	1.5%
	Jidong County Resident	315	6.4%
	Mishan City Employee	38	0.8%
	Mishan City Resident	247	5.0%
	Hulin City Employee	57	1.2%
	Hulin City Resident	146	3.0%
	Self-pay	429	8.7%
	Non-local Insurance	99	2.0%
	State Farm Insurance	45	0.9%
	Railway Insurance	8	0.2%
Regional distribution			Hospitalization rate per 100,000
	Jiguan District	2,254	380.1
	Chengzihe District	333	624.5
	Didao District	293	518.9
	Hengshan District	365	687.2
	Lishu District	209	433.6
	Mashan District	64	914.3
	Jidong County	396	286.4
	Mishan City	318	207.8
	Hulin City	246	178.2
	External Regions	57	—

### 3.2 Age distribution

**Figure 2:**
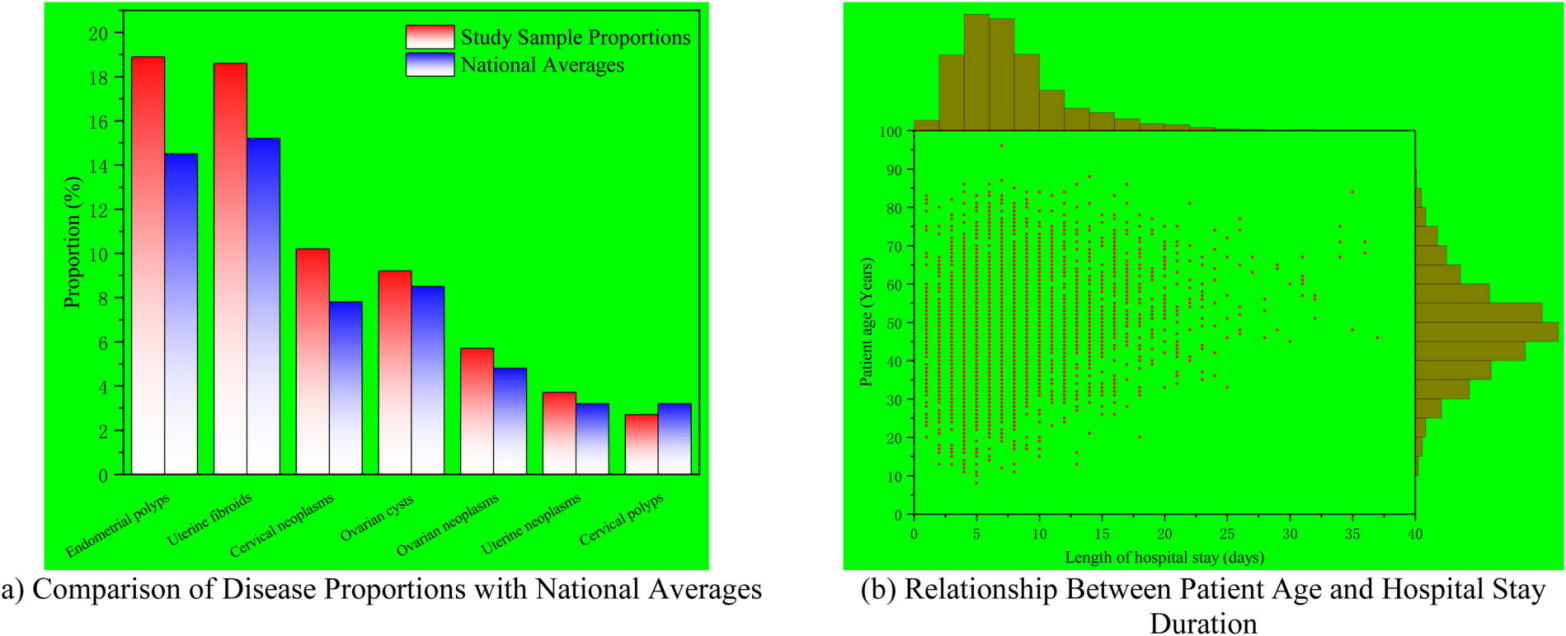
Age-stratified comparative analysis of gynecological disease profiles and healthcare utilization patterns in northeastern mining regions versus national benchmarks. **(a)** Comparison of disease proportions with national averages. **(b)** Relationship between patient age and hospital stay duration.

Patient ages ranged from 8 to 92 years, with a mean age of 47.2 ± 12.8 years and median of 46 years. The age distribution exhibited a unimodal pattern with rightward skew, peaking in the 40–49 age group (Figure 2). The majority of patients (60.7%) were aged 40–59 years, with the 40–49 age group comprising the largest proportion (1,669 cases, 33.8%), followed by the 50–59 age group (1,327 cases, 26.9%) and the 30–39 age group (857 cases, 17.4%). Together, these three age groups accounted for 78.1% of all admissions. Adolescent patients (8–19 years) represented only 1.3% of cases, while elderly patients aged 80 years and above constituted 1.0%. Notably, 15.7% of patients were aged 60 years or older, highlighting the importance of geriatric gynecological care in this population.

Disease spectra varied significantly across age groups (χ^2^ = 186.43, *P* < 0.001). In adolescents (8–19 years), the predominant diagnoses were congenital reproductive tract anomalies (39.4%) and vulvar injuries (24.2%). Among young adults (20–29 years), ectopic pregnancy was the leading diagnosis (28.8%). Uterine fibroids predominated in women aged 30–49 years (42.3%). Malignancy rates increased progressively with age, from 6.2% in the 40–49 age group to 18.7% in the 60–69 age group (*P* < 0.001), representing a 3-fold increase.

### 3.3 Disease composition

#### 3.3.1 Overall disease profile

Uterine pathologies constituted the largest disease category, accounting for 2,842 cases (57.6%), followed by ovarian diseases (787 cases, 15.9%) and cervical diseases (667 cases, 13.5%). These three categories collectively represented 87.0% of all admissions. Less common categories included fallopian tube diseases (3.5%), ectopic pregnancy (2.3%), pelvic disorders (1.8%), vaginal disorders (1.5%), and abortion-related conditions (0.9%). Detailed disease composition is presented in Table 2.

**Table 2:** Disease composition of gynecological inpatients (*n* = 4,935).

Disease category	Subcategory	Cases (*n*)	Proportion (%)	National average (%)	*χ* ^2^	*P*-value
Uterine diseases		2,842	57.6	52.3	18.72	<0.001
	Endometrial polyps	933	18.9	14.5	24.61	<0.001
	Uterine fibroids	920	18.6	15.2	15.38	<0.001
	Uterine malignancies	185	3.7	3.2	1.86	0.173
	Endometrial thickening	146	3.0	2.8	0.42	0.517
	Endometrial hyperplasia	143	2.9	2.4	2.31	0.129
	Uterine prolapse	115	2.3	1.8	3.12	0.077
	Abnormal uterine bleeding	99	2.0	2.2	0.38	0.538
	Other uterine disorders	301	6.1	5.9	0.14	0.708
Ovarian diseases		787	15.9	14.2	4.28	0.039
	Ovarian cysts	455	9.2	8.5	1.23	0.267
	Ovarian neoplasms	283	5.7	4.8	3.42	0.064
	Other ovarian disorders	49	1.0	0.9	0.18	0.671
Cervical diseases		667	13.5	11.8	5.16	0.023
	Cervical neoplasms	503	10.2	7.8	13.86	<0.001
	Cervical polyps	135	2.7	3.2	1.62	0.203
	Other cervical disorders	29	0.6	0.8	1.08	0.299
Fallopian tube diseases		173	3.5	3.8	0.52	0.471
Ectopic pregnancy		114	2.3	2.5	0.38	0.538
Pelvic disorders		89	1.8	2.1	0.86	0.354
Vaginal disorders		73	1.5	1.8	1.12	0.290
Abortion-related		43	0.9	1.2	1.68	0.195
Other		147	3.0	3.2	0.28	0.597

National averages derived from National Women's Health Statistical Yearbook (2020–2023).

#### 3.3.2 Comparison with national averages

Compared with national data from tertiary hospitals, several disease categories showed significantly different proportions in our study population (Table 2). The overall proportion of uterine diseases was significantly higher than the national average (57.6% vs. 52.3%, *χ*^2^ = 18.72, *P* < 0.001). Specifically, endometrial polyps (18.9% vs. 14.5%, *χ*^2^ = 24.61, *P* < 0.001) and uterine fibroids (18.6% vs. 15.2%, *χ*^2^ = 15.38, *P* < 0.001) were significantly more prevalent. Ovarian diseases (15.9% vs. 14.2%, *χ*^2^ = 4.28, *P* = 0.039) and cervical diseases (13.5% vs. 11.8%, *χ*^2^ = 5.16, *P* = 0.023) were also significantly elevated, with cervical neoplasms showing a particularly marked difference (10.2% vs. 7.8%, *χ*^2^ = 13.86, *P* < 0.001). Figure 2a provides a visual comparison of disease proportions between the study population and national averages.

### 3.4 Regional hospitalization patterns

Hospitalization rates varied substantially across the nine administrative regions (Table 1, Figure 2b). The highest hospitalization rates were observed in Mashan District (914.3 per 100,000 population), Hengshan District (687.2 per 100,000), and Chengzihe District (624.5 per 100,000). The lowest rates were observed in Hulin City (178.2 per 100,000) and Mishan City (207.8 per 100,000). These regional differences were statistically significant (*χ*^2^ = 312.56, *P* < 0.001).

When comparing mining-intensive districts (Mashan, Hengshan, Chengzihe, and Didao) with other areas, the aggregate hospitalization rate was 2.2-fold higher in mining-intensive regions (661.2 vs. 297.2 per 100,000; *χ*^2^ = 186.72, *P* < 0.001). Patients from mining-intensive districts (*n* = 1,055) also showed different disease profiles, with higher proportions of uterine fibroids (22.4% vs. 17.6%, *χ*^2^ = 12.38, *P* < 0.001), cervical precancerous lesions (8.7% vs. 5.2%, *χ*^2^ = 16.84, *P* < 0.001), and vaginitis (3.2% vs. 1.8%, *χ*^2^ = 7.62, *P* = 0.006) compared to patients from other districts.

### 3.5 Length of hospital stay

The mean length of hospital stay was 6.8 ± 4.2 days (median: 6 days; interquartile range: 4–9 days). The majority of patients (66.2%) had stays of 3–8 days, with the 3–5 day range being most common (35.1%), reflecting the predominance of laparoscopic surgical procedures. Short stays (1–2 days) accounted for 5.1% of cases, while extended stays (≥15 days) comprised 7.8%.

Length of stay varied significantly by disease category (*F* = 86.72, *P* < 0.001). Patients with malignancies had the longest stays (12.6 ± 6.8 days), followed by pelvic inflammatory disease (8.2 ± 4.1 days) and uterine disorders requiring open surgery (6.4 ± 3.8 days). Abortion-related admissions had the shortest stays (3.2 ± 1.6 days).

Segmented regression analysis revealed a nonlinear relationship between age and length of stay (Figure 2). In patients under 50 years, age was not significantly associated with hospitalization duration. In patients aged 50–65 years, each additional year of age corresponded to a 0.11-day increase in length of stay (*β* = 0.11, 95% CI: 0.08–0.14, *P* < 0.001). In patients over 65 years, the relationship became steeper, with accelerated prolongation of hospital stays reflecting the complexity of care in elderly patients.

Using Origin for segmented regression analysis, the mathematical relationship between age (*X*) and hospital stay (*Y*) was modeled as follows:

Equation (1): Base model for age–hospitalization relationship:

$$\ln(Y+1)=\beta_{0}+\beta_{1}(X-50)+\beta_{2}(X-65{)}^{2}+\varepsilon$$ (1)

Equation (2): Age-stratified refinement:

$$Y=\{\begin{array}{ccc} 3.82+0.11X & 50\leq X\leq 65 & \beta =0.21,P<0.001\\ 4.79+0.27(X-65)+0.04{\left(X-65\right)}^{2} & X>65 & \beta =0.18,P=0.009 \end{array}$$ (2)

In Equations (1) and (2), *Y* = length of hospital stay (days); *X* = patient age (years); *β*₀ = intercept; *β*₁ and *β*_2_= age coefficient; and *ε* = error term.

#### 3.5.1 Key findings

Patients aged <50 years: No statistically significant association was observed between age and length of hospital stay, aligning with the physiological stability profile of younger cohorts.

Patients aged 50–65 years: A linear age-dependent prolongation was identified, with each additional year of age corresponding to a 0.11-day increase in hospitalization duration, consistent with declining physiological resilience patterns documented in middle-aged populations.

Patients aged >65 years: A significant nonlinear acceleration effect emerged, reflecting compounded risks from comorbidities prevalent in geriatric cohorts. This quadratic trajectory aligns with national data showing accelerated care demands in elderly surgical patients.

### 3.6 Insurance coverage and healthcare access

Insurance coverage was high, with 91.3% of patients (4,506/4,935) having some form of medical insurance. Employee insurance (41.7%) and urban–rural resident insurance (46.7%) were the predominant types. Self-pay patients (429 cases, 8.7%) demonstrated significantly longer delays from symptom onset to hospital presentation (8.2 ± 5.6 months vs. 3.4 ± 2.8 months for insured patients; *t* = 16.28, *P* < 0.001) and higher proportions of advanced-stage cervical disease at presentation (Stage II or higher: 42.3% vs. 18.6%; *χ*^2^ = 28.64, *P* < 0.001).

## 4 Discussion

### 4.1 Principal findings

This cross-sectional study of 4,935 gynecological inpatients at a tertiary hospital in northeastern China's Jixi mining region revealed several notable epidemiological patterns. First, hospitalizations were concentrated among perimenopausal women aged 40–59 years, consistent with the hormonal and physiological changes of this life stage, which predispose to uterine pathologies. Second, uterine diseases, particularly endometrial polyps and uterine fibroids, were the predominant conditions, with proportions significantly exceeding national averages. Third, substantial geographic variation in hospitalization rates was observed, with mining-intensive districts showing rates more than double those of other areas. Fourth, the relationship between age and length of stay was nonlinear, with accelerated prolongation in elderly patients. These findings have implications for healthcare planning and nursing practice in similar resource-dependent regions.

### 4.2 Comparison with existing literature

The concentration of gynecological hospitalizations in the 40–59 age group aligns with previous reports from both China and international studies (13, 14). This pattern reflects the well-established associations between perimenopausal estrogen fluctuations and conditions including uterine fibroids, endometrial polyps, and dysfunctional uterine bleeding (15). The predominance of uterine pathologies is also consistent with national survey data identifying uterine fibroids as the most common gynecological condition in Chinese women (16).

However, the proportions of certain conditions in our study population exceeded national averages. Endometrial polyps (18.9% vs. 14.5%), uterine fibroids (18.6% vs. 15.2%), and cervical neoplasms (10.2% vs. 7.8%) were all significantly elevated. These differences may reflect regional variations in disease prevalence, healthcare-seeking behavior, screening practices, or healthcare access patterns. Similar regional variations have been reported in other Chinese provinces (17) and internationally (18).

The elevated hospitalization rates in mining-intensive districts represent a key finding requiring cautious interpretation. While the 2.2-fold higher hospitalization rate in these areas is striking, this study cannot determine whether this reflects higher disease prevalence, differences in healthcare-seeking behavior, variations in local healthcare capacity, or other factors. Previous studies have suggested that populations in mining regions may face elevated health risks due to environmental contamination and socioeconomic factors (19, 20), but our study design did not permit direct assessment of these potential mechanisms.

### 4.3 Potential explanatory factors

Several factors may contribute to the observed patterns, though our data do not permit causal inference. Geographic disparities in hospitalization rates may reflect differences in healthcare infrastructure, with patients in remote mining districts potentially having limited access to primary care and thus presenting for hospital admission at later disease stages. This interpretation is supported by our finding that mining-area patients had higher proportions of advanced cervical lesions. Alternative explanations include true differences in disease prevalence related to environmental factors, occupational exposures among residents, or demographic differences between regions.

It should be noted that the hospitalization rate calculations are subject to denominator uncertainty, as the census population may not precisely reflect the hospital's actual service population. Differential healthcare-seeking patterns across districts—with some residents seeking care elsewhere—could contribute to the observed geographic variations independently of true disease prevalence differences.

The elevated proportions of uterine pathologies compared to national averages may relate to demographic factors (population age structure), environmental factors, lifestyle patterns, or regional screening practices. Studies from other regions have suggested associations between industrial exposures and reproductive health outcomes (21–23), but our data cannot evaluate these potential associations.

### 4.4 Implications for nursing practice

The epidemiological patterns identified have several implications for nursing care in this region. The concentration of hospitalizations among perimenopausal women suggests the need for age-specific nursing approaches, including attention to menopausal symptoms, fertility concerns in younger patients with uterine fibroids, and recognition of malignancy risk in older patients. The high proportion of patients requiring surgical intervention (reflected in the 3–8-day modal length of stay consistent with laparoscopic procedures) highlights the importance of evidence-based perioperative nursing care.

For patients from mining-intensive districts, the higher rates of advanced disease presentations suggest potential benefits from enhanced health education and screening outreach. Nursing-led interventions could include community education programs targeting early symptom recognition and healthcare navigation assistance for populations with limited healthcare access. The finding that self-pay patients had delayed presentations and more advanced disease highlights the importance of social work integration and financial screening in nursing assessments.

The nonlinear relationship between age and length of stay—with acceleration in patients over 65 years—underscores the growing importance of geriatric competencies in gynecological nursing as populations continue to age. This finding aligns with studies demonstrating increased perioperative complexity and complication rates in elderly surgical patients (24).

### 4.5 Strengths and limitations

This study has several strengths. The large sample size (*n* = 4,935) provides robust estimates of disease proportions and hospitalization patterns. The 5-year study period captures temporal stability of findings. The inclusion of all eligible patients without sampling reduces selection bias. Comparison with national data provides context for interpreting regional patterns.

However, several important limitations must be acknowledged. First, this single-center study from one tertiary hospital may not be representative of all gynecological disease burdens in the region, as patients with mild conditions managed in primary care or those who did not seek care are not captured. Second, reliance on medical records limited the variables available for analysis. Critically, we did not have data on occupational history, duration of residence, specific environmental exposures, lifestyle factors, or detailed reproductive history. Therefore, we cannot directly assess whether the elevated hospitalization rates in mining districts reflect occupational or environmental exposures versus other factors such as healthcare access patterns or demographic differences. Third, the comparison with national data involved published aggregate statistics from different time periods, introducing potential temporal heterogeneity. Fourth, the retrospective design precludes causal inference regarding observed associations. Fifth, ICD-10 coding accuracy was not independently validated, and coding practices may have varied over the study period.

Future research should employ prospective designs with comprehensive exposure assessment, include multiple centers for broader generalizability, and directly measure potential environmental or occupational factors to elucidate mechanisms underlying regional variations in disease patterns.

### 4.6 Clinical nursing implications

The epidemiological patterns identified in this study inform several recommendations for nursing practice in this region. These recommendations are derived from the observed disease patterns and patient characteristics, integrated with established evidence-based nursing principles.

#### 4.6.1 Rationale for nursing framework development

The development of targeted nursing strategies is justified by the distinct patterns observed in this population. The concentration of hospitalizations among perimenopausal women (40–59 years), the predominance of uterine pathologies requiring surgical intervention, the geographic disparities suggesting delayed presentations in mining districts, and the vulnerability of uninsured patients all indicate the need for tailored nursing approaches rather than uniform care protocols.

#### 4.6.2 Age-stratified care approaches

Based on the observed age distribution and age-specific disease patterns, a three-tiered framework may be considered. For adolescent patients (representing 1.3% of admissions, primarily with congenital anomalies), nursing priorities include developmentally appropriate communication, privacy protection, and family engagement (25). For reproductive-age women (20–49 years, comprising the majority of patients with uterine fibroids and ectopic pregnancy), key considerations include fertility preservation counseling, enhanced recovery protocols for minimally invasive surgery, and postoperative reproductive health education (26). For perimenopausal and elderly patients (≥50 years, with increasing malignancy rates), nursing priorities include symptom recognition education, thromboprophylaxis implementation, management of comorbidities, and psycho-oncology support when indicated (27).

#### 4.6.3 Disease-specific care considerations

For the predominant conditions (uterine fibroids, endometrial polyps, and cervical neoplasms), standardized care pathways incorporating preoperative assessment, patient education regarding surgical approaches, early mobilization, and appropriate discharge planning are warranted. For patients with malignancies, comprehensive care addressing treatment-related symptoms, psychological support, and continuity of care is essential (28).

#### 4.6.4 Addressing regional disparities

The elevated hospitalization rates and more advanced disease presentations in mining-intensive districts suggest potential benefits from community outreach and health education initiatives. Nursing-led programs could address symptom recognition, screening importance, and healthcare navigation. Collaboration between hospital nursing departments and community health services may facilitate earlier presentation and improved outcomes (29).

#### 4.6.5 Quality metrics

Monitoring of nursing quality indicators—including perioperative complication rates, patient satisfaction, health education effectiveness, and care continuity measures—can support ongoing quality improvement aligned with the specific needs of this population (30).

### 4.7 Study limitations

This study has several important limitations that should be considered when interpreting the findings.

First, this is a single-center study conducted at one tertiary hospital in Jixi City, which may limit generalizability to other settings. As a tertiary referral center, our hospital likely receives a disproportionate number of complex and severe cases requiring specialized care or surgical intervention, while milder conditions managed in primary care facilities or outpatient settings are not captured. This referral bias means that the disease proportions and severity profiles observed in our study may not accurately reflect the true burden of gynecological diseases in the general population. Moreover, women who did not seek medical care due to financial constraints, geographic barriers, or lack of symptom awareness are not represented in our data. Therefore, our findings should be interpreted as characterizing hospitalization patterns at a tertiary facility rather than population-level disease prevalence.

Second, the retrospective design relying on medical records limited the variables available for analysis. Critically, we did not collect data on occupational history or specific workplace exposures, duration of residence at current address, direct measures of environmental exposure (such as air or water quality at residential locations), lifestyle factors (including diet, physical activity, and smoking status), detailed reproductive history, or HPV vaccination and screening history. Consequently, this study cannot directly evaluate whether the elevated hospitalization rates in mining districts reflect occupational or environmental exposures, socioeconomic factors, healthcare access patterns, or other mechanisms.

Third, hospitalization rates calculated using Seventh National Census population data as denominators should be interpreted with caution. Census figures may not accurately represent the hospital's actual catchment population for several reasons: (a) Population mobility and migration patterns may have changed since the 2020 census. (b) Healthcare-seeking behavior varies across districts, with some residents potentially seeking care at other facilities or in other cities. (c) Our hospital, as the main tertiary referral center, may attract patients from beyond its nominal catchment area while simultaneously losing some local patients to facilities in larger cities. (d) Finally, the proportion of female residents eligible for gynecological care (excluding very young children) varies across districts. These factors may lead to either overestimation or underestimation of true hospitalization rates in different districts. The regional comparisons should therefore be viewed as hypothesis-generating rather than definitive measures of disease burden.

Finally, this study included only inpatient admissions and did not capture emergency department visits or outpatient encounters, which may have different disease profiles.

## 5 Conclusions

This cross-sectional study of 4,935 gynecological inpatients at a tertiary hospital in northeastern China's Jixi region revealed several notable patterns. Hospitalizations were concentrated among perimenopausal women aged 40–59 years, with uterine pathologies (particularly endometrial polyps and uterine fibroids) representing the predominant diagnoses at proportions exceeding national averages. Substantial geographic variation was observed, with mining-intensive districts showing hospitalization rates more than double those of other areas and higher proportions of advanced disease presentations. Self-pay patients demonstrated delayed healthcare-seeking behavior and more advanced disease staging. These findings provide foundational epidemiological data on gynecological hospitalization patterns at a tertiary facility in an understudied region, offering insights for healthcare planning and nursing practice development, such as the consideration of age-stratified care, enhanced outreach to underserved areas, and focused attention to financially vulnerable populations.

However, as a hospital-based study capturing primarily cases requiring inpatient care, the results should not be extrapolated to represent population-level disease prevalence. Furthermore, the mechanisms underlying the observed regional variations—whether related to environmental factors, healthcare access, or other determinants—remain unclear and warrant further investigation through prospective studies with comprehensive exposure assessment.

## Data Availability

The original contributions presented in the study are included in the article/supplementary material, further inquiries can be directed to the corresponding author.
